# QTL Mapping and Marker Development for Tolerance to Sulfur Phytotoxicity in Melon (*Cucumis melo*)

**DOI:** 10.3389/fpls.2020.01097

**Published:** 2020-07-22

**Authors:** Sandra E. Branham, James Daley, Amnon Levi, Richard Hassell, W. Patrick Wechter

**Affiliations:** ^1^ U.S. Vegetable Laboratory, Agricultural Research Service, U.S. Department of Agriculture, Charleston, SC, United States; ^2^ Coastal Research and Education Center, Clemson University, Charleston, SC, United States; ^3^ HM.CLAUSE, Davis, CA, United States

**Keywords:** melon, *Cucumis melo*, sulfur tolerance, quantitative trait loci mapping, Kompetitive Allele-Specific PCR, sulfur phytotoxicity, whole genome resequencing

## Abstract

Elemental sulfur is an effective, inexpensive fungicide for many foliar pathogens, but severe phytotoxicity prohibits its use on many melon varieties. Sulfur phytotoxicity causes chlorosis and necrosis of leaf tissue, leading to plant death in the most sensitive lines, while other varieties have little to no damage. A high-density, genotyping-by-sequencing (GBS)-based genetic map of a recombinant inbred line (RIL) population segregating for sulfur tolerance was used for a quantitative trait loci (QTL) mapping study of sulfur phytotoxicity in melon. One major (qSulf-1) and two minor (qSulf-8 and qSulf-12) QTL were associated with sulfur tolerance in the population. The development of Kompetitive Allele-Specific PCR (KASP) markers developed across qSulf-1 decreased the QTL interval from 239 kb (cotyledons) and 157 kb (leaves) to 97 kb (both tissues). The markers were validated for linkage to sulfur tolerance in a set of melon cultivars. These KASP markers can be incorporated into melon breeding programs for introgression of sulfur tolerance into elite melon germplasm.

## Introduction

Elemental sulfur is widely used as an organic fungicide in fruit and vegetable crops for control of powdery mildew and rusts (Williams and Cooper, 2004). For cucurbits, sulfur is an inexpensive and effective method for controlling powdery mildew (*Podosphaera xanthii*) (Koller, 2010; Keinath and Dubose, 2012). Sulfur can be applied to plants by direct contact, diffusion through water, or as a vapor (Bent, 1967). The underlying fungicide mechanism of sulfur is not known, but the current hypothesis is that it permeates into the fungus and interferes with mitochondrial respiration (Cooper and Williams, 2004), resulting in the inhibition of conidial germination (Gogoi et al., 2013). The Fungicide Resistance Action Committee defines sulfur’s mode of action as multi-site contact activity and is considered a low risk for pathogen resistance development.

Although sulfur is used on many cucurbits, including melon, phytotoxic reaction to sulfur can range from extremely sensitive resulting in death of the plant, to completely tolerant (Johnson and Mayberry, 1980; Perchepied et al., 2004; Gogoi et al., 2013). Sulfur phytotoxicity is manifested as necrosis and pronounced “burning” on the leaf tissue starting four days after dusting fruiting melon plants in field conditions (Johnson and Mayberry, 1980). In greenhouse conditions, vaporized sulfur causes symptoms in as little as 24 h post-application in highly susceptible melon lines.

The limited research on sulfur phytotoxicity in melon has focused on sulfur dust application for tolerance screening and QTL discovery (Johnson and Mayberry, 1980; Perchepied et al., 2004). A sulfur tolerance screen of 31 melon cultivars by Johnson and Mayberry (1980) described 23 cultivars as tolerant and 8 as susceptible. In another study, 236 melon accessions from around the world were screened for response to sulfur, with 47% exhibiting complete tolerance (Perchepied et al., 2004). Perchepied et al. (2004) successfully mapped one major and two minor QTL associated with sulfur tolerance in two recombinant inbred line (RILs) populations sharing a common tolerant parent. The sulfur tolerance allele (contributed by the tolerant parent ‘Vedrantais’) of the major QTL exerted complete dominance when crossed to PI124112 and incomplete dominance to PI161375. The two minor QTL were only detected in the Vedrantais × PI124112 population. Perchepied et al. (2004) used a previously published genetic map (Périn et al., 2002) that was limited by low marker density (460 markers) resulting in poor QTL resolution, with the major QTL spanning 21 cM. The physical position of the QTL was not reported as there was not yet a melon reference genome available.

In this study, we utilized the high-density, genome-anchored genetic map available for the MR-1 × AY RIL population (Branham et al., 2018) to identify QTL associated with tolerance to vaporized sulfur. In addition, PCR-based markers for the major QTL were developed and tested in the population and various elite germplasm for linkage to sulfur tolerance and should be useful to breeders utilizing a marker assisted breeding scheme to increase the efficiency of sulfur tolerance introgression into elite cultivars.

## Methods

### Experimental Design

**Figure 1:**
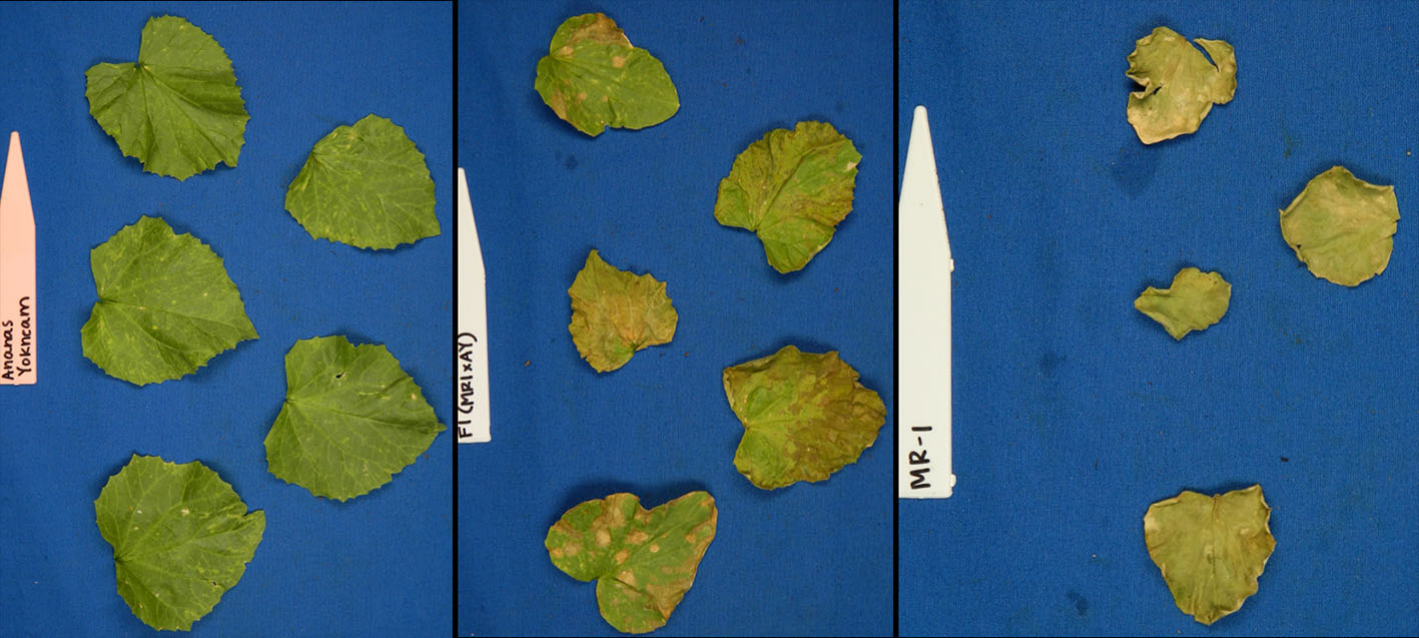
Photographs of young plants of the sulfur tolerant (AY) and sensitive (MR-1) parents after vaporized elemental sulfur treatment.

**Figure 2:**
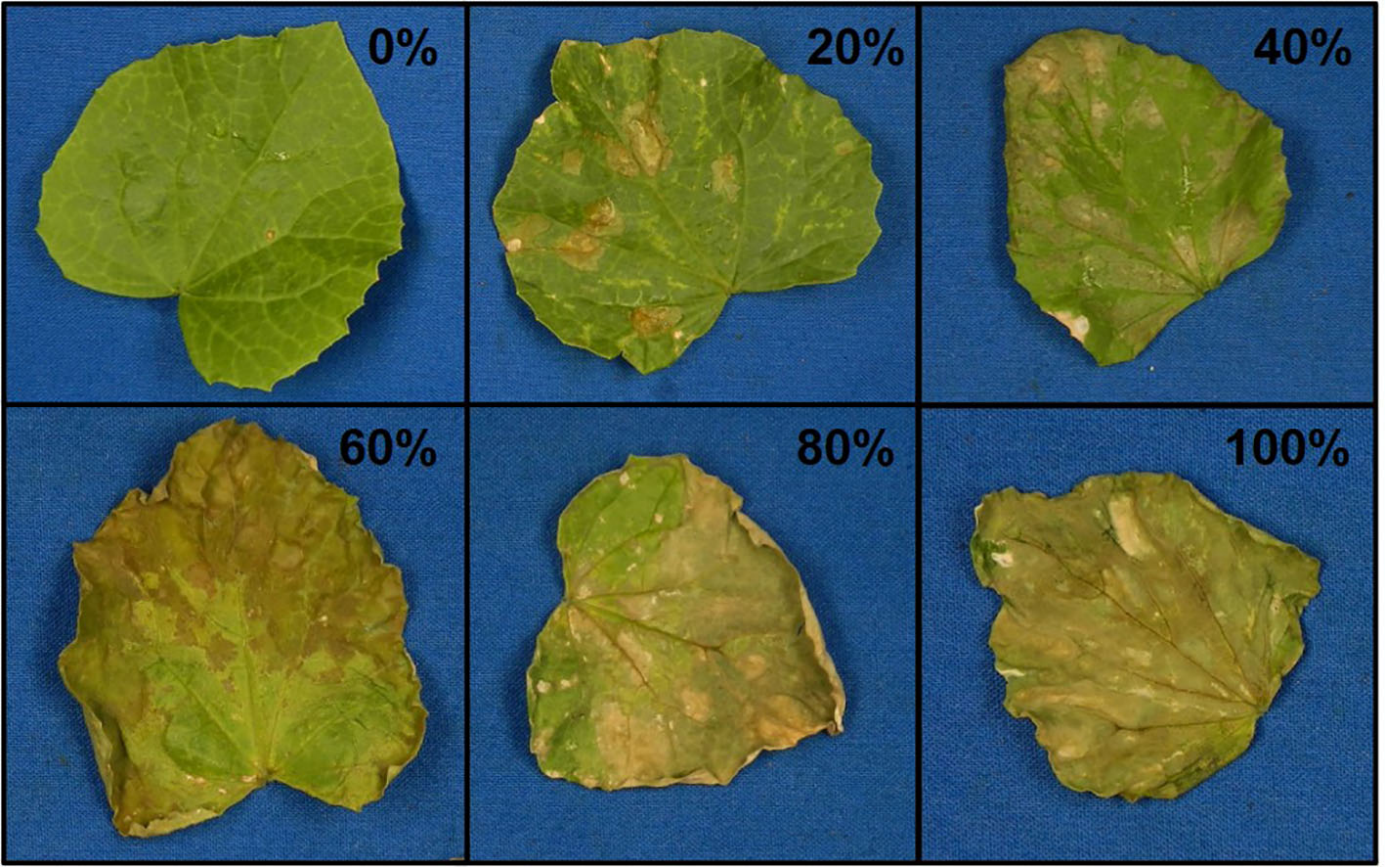
Representative photos of damage ratings (0–100% in 20% increments) of melon leaves after vaporized elemental sulfur treatment.

A previously described RIL population (Branham et al., 2018) consisting of 170 lines generated from a cross of MR-1 and Ananas Yok’neum (AY) was evaluated for elemental sulfur tolerance. The Israeli cantaloupe cultivar Ananas Yok’neum was the sulfur tolerant parent and the inbred *C. melo* line MR-1 (Thomas, 1986) was the sensitive parent (**Figure 1**). Two independent greenhouse tests of the parents and population were initiated in May and June 2017. Each test was planted in a randomized complete block design with two replicates of five plants each. Lines were seeded into Metromix 360 (Sun Gro Horticulture, Agawam, MA) in 50-cell propagation trays (Hummert International, Earth City, MO) and allowed to grow to the 2–3 fully expanded leaf stage in a sulfur-free glass greenhouse. Temperature of the greenhouse were maintained at 30°C ± 5°C. Seedlings were fertilized the day prior to sulfur treatment by soaking trays in a liquid fertilizer solution (3 g Peters water soluble fertilizer per liter) (Scotts, Maryville, OH, USA). Trays were transferred into a temperature-controlled, 650 m^3^ glass greenhouse for sulfur treatment. Temperature of the greenhouse were maintained at 30°C ± 5°C. Elemental sulfur (Soil Sulfur:>99% purity, National Garden Wholesale, Vancouver, WA, USA) was vaporized using two sulfur burners (Wilmod Sulfur Evaporator WSE75; Zoetermeer, Netherlands) for 4 hours nightly. The sulfur burners were ~2 m above the work benches, suspended 0.75 m below a circulation fan. The two burners were on adjacent ends of the greenhouse. Each sulfur burner vaporized approximately 1.2 g of sulfur per night. On the fifth day, lines were evaluated for sulfur tolerance by recording percent necrosis for both the most damaged cotyledon and true leaf on every plant (**Figure 2**). The percent necrosis for each RIL (cotyledon and true leaf) was averaged from evaluations of twenty plants (two tests × two reps × five plants). F_1_ seeds failed to germinate in the original study, so an additional test was performed that included the parents and new seed of the F_1_ hybrid. Two replicates of ten seeds per line were planted in a greenhouse trial in June 2018. An additional test of thirty melon accessions (cultivars and PIs) was evaluated to test the utility of the sulfur markers in a variety of germplasm. Two replicates of five seeds each were planted in a greenhouse trial in March 2019. These additional studies followed the same protocols described above.

### Statistical Analysis

Pearson’s correlation (r) of line means between tests and between tissue types was calculated with the stats package of R version 3.4.1 (R Core Team, 2018). Broad-sense heritability (H^2^) of sulfur tolerance, measured as percent affected leaf area (chlorosis and/or necrosis), was determined separately for each tissue type as the RIL variance divided by the total variance in percentage affected tissue area using variance components estimated with a linear mixed model in ASReml-R v3.0 (Gilmour et al., 2009). The model included RIL, test, interaction of RIL and test, replicate, and tray nested within test as random effects.

### QTL Mapping

We used the previously published (Branham et al., 2018) high-density genetic map developed for this population for all QTL mapping analyses, which included 5,663 imputed, binned SNPs across the 12 chromosomes (=linkage groups) of the *C. melo* genome (Garcia-Mas et al., 2012). Haley–Knott regression (Haley and Knott, 1992) was used for multiple QTL mapping (MQM) with the stepwiseqtl function (Zeng et al., 1999; Broman and Speed, 2002; Broman and Sen, 2009) of Rqtl (Broman et al., 2003). The optimal QTL model based upon penalized LOD score (Manichaikul et al., 2009) was chosen through an automated forward and backward search algorithm. One thousand permutations of a two-dimensional, two QTL scan were used to calculate penalties and the genome-wide significance threshold for QTL detection. Multiple QTL models were visualized through LOD profile plots generated from forward selection using standard interval mapping with Haley–Knott regression (Haley and Knott, 1992). Distributions of necrosis percentage of both cotyledons and true leaves did not meet the assumptions of parametric interval mapping, therefore the non-parametric model of the scanone function (Kruskal and Wallis, 1952; Kruglyak and Lander, 1995) was used for QTL verification. Genes within the 1.5-LOD interval of the major QTL were identified using the functional annotation of the *C. melo* reference genome v3.5.1 (Garcia-Mas et al., 2012), which was obtained through batch query at http://cucurbitgenomics.org/ (Zheng et al., 2019). In addition to using the functional annotation provided with the reference genome to search for candidate genes, conserved domains of genes were identified using the National Center for Biotechnology Information’s batch CD search (CDDv3.16 database) (Marchler-Bauer and Bryant, 2004; Marchler-Bauer et al., 2011; Marchler-Bauer et al., 2015; Marchler-Bauer et al., 2017).

### Parental Resequencing

Genomic DNA was extracted from young leaf tissue of both parental lines (MR-1 and AY) using a DNeasy Plant Mini kit (Qiagen, Venlo, Netherlands) and sent to the Roy J. Carver Biotechnology Center at the University of Illinois at Urbana-Champaign for whole-genome resequencing. A Hyper Library construction kit (Kapa Biosystems, Roche, Basel, Switzerland) was used to prepare shotgun libraries for each parental DNA. Libraries were quantified by qPCR, pooled, and sequenced on one lane of a NovaSeq 6000 (Illumina, San Diego, CA) with a NovaSeq S2 reagent kit. Paired-end reads (150 bp) were demultiplexed with bcl2fastq v2.20 Conversion software (Illumina). Adaptors were trimmed from the 3’ end of the reads. Duplicated read pairs were removed with perl scripts (https://github.com/Sunhh/NGS_data_processing/blob/master/drop_dup_both_end.pl). Low quality reads were removed with trimmomatic v0.38 (Bolger et al., 2014). The remaining high-quality reads were aligned to *C. melo* reference genome v3.5.1 (Garcia-Mas et al., 2012) with BWA v0.7.17 (Li and Durbin, 2009). Picard v2.18.7 (http://broadinstitute.github.io/picard) was used to assign reads to a read group, tag reads originating from a single DNA fragment, and to create a reference sequence dictionary. The reference genome was indexed with Samtools v0.1.8 (Li et al., 2009). The Genome Analysis Toolkit (GATK v3.6) was used for SNP calling following the best practices for variant discovery (McKenna et al., 2010; Depristo et al., 2011, Van der Auwera et al., 2013). SNPs were filtered with Vcftools v0.1.15 (Danecek et al., 2011) to remove those with any missing data, heterozygous genotypes for either inbred parent, and/or genotype quality score of less than 30. SNPs within the major QTL region were functionally annotated with ANNOVAR version 2017 Jul 16 (Wang et al., 2010). Genes with missense or nonsense mutations and mutations to the promotor (less than 1 kb upstream of the start codon) were considered candidate genes.

### Marker Development

The parental whole-genome resequencing data was used to design markers to saturate the region of the major sulfur tolerance QTL. Eighteen SNPs from across the major QTL region were developed into KASP markers (**Supplementary Table S1**) using “KASP™ by design” services from LGC Genomics (Teddington, Middlesex, UK). PCR reactions (5 µl volume) consisted of 0.07 µl of primer mix (LGC Genomics; fluorophore-labeled allele-specific forward primers and a reverse primer), 2.5 µl of 2× master mix (LGC Genomics) and 20 ng of sample DNA. A standard thermal cycler was used for a touchdown PCR reaction with a 94°C hot-start activation step for 15 min, then ten cycles of 94°C (20 s) and a starting annealing temperature of 61°C that dropped by 0.6°C each cycle. Twenty-six additional cycles of 94°C for 20 s and 55°C for 60 s followed the touchdown steps. Fluorescence was quantified with a Stratagene Mx3005P (Agilent Technologies, Santa Clara, CA) quantitative PCR system at 25°C. Fluorescence values were used to cluster samples into genotypes with MxPro v4.10 software associated with the qPCR machine. Marker linkage to sulfur tolerance in the RIL population was assessed through QTL mapping both alone (KASP markers only) and combined with the binned GBS SNPs following the same procedures as described above. Thirty accessions (cultivars and plant introductions) were evaluated for sulfur tolerance and genotyped with the KASP markers. Correlation between the markers and sulfur phenotype of the accessions was assessed through analyses of variance (ANOVA) with the aov function (Chambers et al., 1992) in R.

## Results

### Elemental Sulfur Tolerance

**Figure 3:**
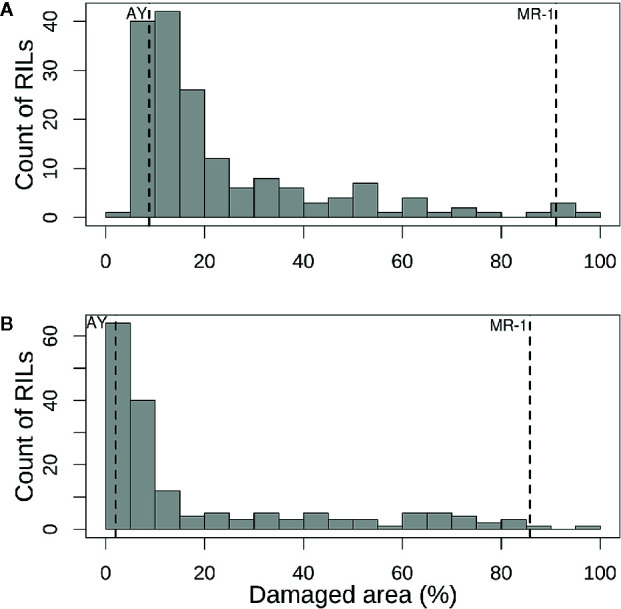
Histograms of mean percentage of damaged (chlorotic or necrotic) area in **(A)** cotyledons and **(B)** leaves of the melon recombinant inbred line population after vaporized elemental sulfur treatment. Means of the tolerant parent (AY) and sensitive parent (MR-1) are indicated by vertical dashed lines.

The population distribution of response to sulfur vapor, although strongly skewed towards tolerance with population means of 23.3 and 19.1% for cotyledons and leaves, respectively (**Figure 3**), varied widely across the population from 5.3 to 100% damage for cotyledons and 1 to 99.5% for leaves (**Supplementary Table S2**). The parents of the population had line means in the expected extremes of the distributions for both tissue types (**Figure 3**). AY had sulfur-induced damage of 8.8% for cotyledons and 2% for leaves, while MR-1 means were 91.5 and 85.4%, respectively. The RIL population means of affected area (chlorotic and/or necrotic) in response to sulfur treatment were highly correlated (p <2.2 × 10^−16^; r = 0.85) between cotyledons and leaves. Correlation between tests was highly significant for both tissue types, but stronger for leaf tissue (p <2.2 × 10^−16^; r = 0.83) than cotyledons (p <2.2 × 10^−16^; r = 0.72). H^2^ was moderate for both tissue types at 0.47 for cotyledons and 0.59 for leaves. An independent test of the parents and F_1_ suggested dominant inheritance of sulfur sensitivity in the cotyledons (MR-1 = 97.8%, AY = 18.0% and F_1_ = 93.0%), but incomplete dominance in the leaves (MR-1 = 90.3%, AY = 2.8% and F_1_ = 75.1%). Mean percentage affected leaf area was higher in all tests and in all samples (parents, F_1_, RILs) for cotyledons than for leaf tissue (**Table 1**).

**Table 1:** Sulfur tolerance means (percent affected tissue; cotyledon and leaf) within and across tests from two studies, one including the parents and RIL population (RILs) and an independent study of the parents and F_1_ hybrid (F_1_).

Study	Experimental unit	Tissue	RIL population mean^a^	AY	MR-1	F_1_^b^
RILs	Across	Cotyledon	23.4	8.8	91.1	NA
RILs	Test 1	Cotyledon	23.5	9.5	90.1	NA
RILs	Test 2	Cotyledon	23.4	8.0	92.2	NA
F_1_	Across	Cotyledon	NA	18.0	97.8	93.1
RILs	Across	Leaf	19.3	2.0	85.8	NA
RILs	Test 1	Leaf	17.0	3.0	90.0	NA
RILs	Test 2	Leaf	21.7	1.0	81.1	NA
F_1_	Across	Leaf	NA	2.8	90.3	75.3

^a,b^NA, Not applicable.

### QTL Mapping

**Figure 4:**
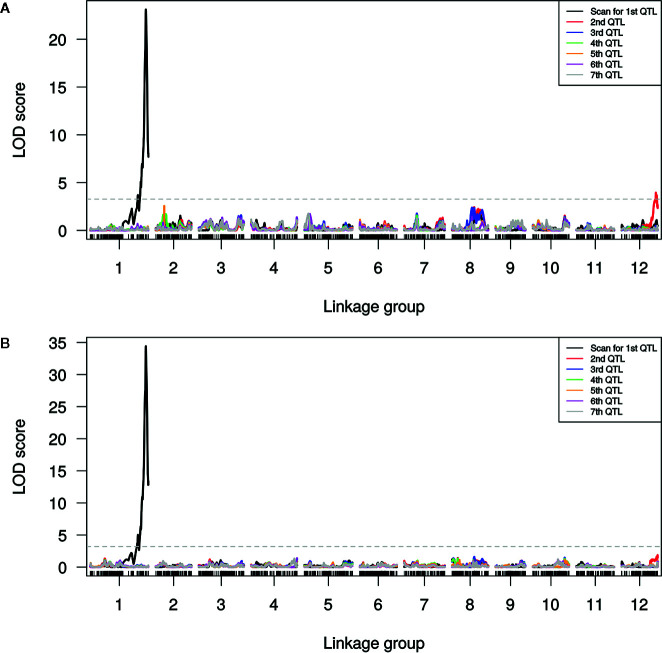
Logarithm of odds (LOD) scores for forward model selection of up to seven QTL associated with mean percentage of damaged (chlorotic or necrotic) area of: **(A)** cotyledons and **(B)** leaves across two replicated greenhouse tests after vaporized elemental sulfur treatment. The initial scan shows the likelihood of the first QTL being located at each SNP in the genome (linkage group = chromosome) with subsequent scans showing the LOD of an additional QTL with the effects of the previous QTL(s) controlled for in the model. The dashed line marks the genome-wide significance threshold.

A single major QTL (qSulf-1) on chromosome 1 explained 56.7 and 60.6% of the variation in mean sulfur tolerance across tests of cotyledons and leaves, respectively (**Figure 4**). RILs homozygous for the AY (tolerant) allele at qSulf-1 had a mean of 6.2% affected leaf area. A second minor QTL (qSulf-12) was associated with mean sulfur tolerance in cotyledons but not leaves (**Table 2**). The major QTL qSulf-1 is epistatic to qSulf-12. RILs homozygous for the AY (tolerant) allele at qSulf-1 show less than 15% damage, regardless of the genotype at qSulf-12 (**Supplementary Figure S1**). The sulfur tolerant allele for both QTL was contributed by the tolerant parent (AY).

**Table 2:** Quantitative trait loci (QTL) associated with sulfur tolerance means (percent affected tissue; cotyledon and leaf) within and across tests in a melon recombinant inbred line (RIL) population using different marker sets.

QTL^a^	Dataset^b^	Tissue	Peak(cM)	1.5-LODInterval (cM)	Total Interval (kb)^c^	LOD	%V_P_^d^	P value	Additive^e^
qSulf-1	Across-KASP	Cotyledon	190.7	190.1–191	97	37.8	62.9	2.0 × 10^−16^	−16.6
	Across		190.5	189.5–191.5	239	32.1	56.7	2.0 × 10^−16^	−15.7
	Test 1		190.5	189–191.8	239	22.1	43.1	2.0 × 10^−16^	−13.3
	Test 2		190.5	189–191.5	239	31.6	56.7	2.0 × 10^−16^	−17.2
	Across-KASP	Leaf	190.7	190.1–191	97	49.5	74.0	2.0 × 10^−16^	−22.3
	Across		190.5	189.6–191.5	157	34.4	60.6	0	−20.3
	Test 1		190	189–191.5	239	20.2	42.1	0	−13.4
	Test 2		190.5	189.6–191.5	157	38.3	65.7	0	−27.1
qSulf-12	Across-KASP	Cotyledon	117.7	117–119	174	12.9	14.7	2.3 × 10^−13^	−7.5
	Across		117.7	116.8–121.4	410	9.9	12.6	2.0 × 10^−10^	−6.6
	Test 1		118	117–119.3	174	9.3	15.0	8.8 × 10^−10^	−7.6
	Across-KASP	Leaf	118	101.5–123.7	2,200	7.2	5.6	9.3 × 10^−8^	−5.4
qSulf-8	Test 2	Cotyledon	68.4	66–69.2	360	8.9	11.3	2.0 × 10^−9^	6.5
q1xq12	Across-KASP	Cotyledon	NA	NA	NA	8.2	8.7	1.3 × 10^−9^	6.5
	Across		NA	NA	NA	6.0	7.2	2.3 × 10^−7^	5.9
	Test 1		NA	NA	NA	5.1	7.9	1.6 × 10^−6^	6.3
	Across-KASP	Leaf	NA	NA	NA	5.1	3.8	2.0 × 10^−6^	−7.1
q1xq8	Test 2	Cotyledon	NA	NA	NA	6.5	7.9	7.4 × 10^−8^	5.2

^a^QTL is named according to: q(trait abbreviation) − (chromosome = linkage group).

Epistatic interaction between qSulf-1 and qSulf-12 = q1xq12.

Epistatic interaction between qSulf-1 and qSulf-8 = q1xq8.

^b^Dataset used for QTL analysis: Means across two tests or means within independent tests; All genotypic data included the GBS SNPs and those listed as -KASP also included KASP markers.

^c^Physical distance of the genome corresponding to the 1.5-LOD interval of the QTL.

^d^Percent of the phenotypic variation explained by the QTL.

^e^Additive effect of the QTL.

MQM of cotyledon and leaf sulfur tolerance means for each test separately (tests 1 and 2) confirmed the location and major contribution (explained 42.1–65.7% of the variation in sulfur tolerance) of qSulf-1 (**Table 2**; **Supplementary Figure S2**). No minor QTL were associated with variation in leaf damage either across or within tests. The minor QTL identified for cotyledon damage, however, varied between the tests. Mean cotyledon damage across tests and in test 1 both identified qSulf-12 and the epistatic interaction with qSulf-1 (**Table 2**; **Supplementary Figure S2**). Sulfur tolerance means for test 2 were instead associated with a new minor QTL, qSulf-8 (**Supplementary Figure S2**). The effect of this QTL was also masked by the AY allele from qSulf-1 but had a negative interaction (**Supplementary Figure S3**). The sulfur tolerant allele for qSulf-8 was contributed by the sulfur sensitive parent (MR-1). The Poisson distribution of sulfur response in the RIL population can be explained by the interaction of the QTL. RILs that were homozygous for the sulfur tolerant allele at qSulf-1 (N = 117 RILs) had mean damage of 14.0 and 6.3% in the cotyledons and leaves, respectively and represent the strong skew towards sulfur tolerance. The remaining tail of the distribution is comprised of individuals homozygous for MR-1 alleles at qSulf-1, with spread of the tail explained by genotypes at the minor QTL. RILs homozygous for sulfur sensitivity alleles at qSulf-1 but sulfur tolerance alleles at one or both of the minor QTL had mean damage of 38.3% for cotyledons and 43.8% for leaves. RILs at the extreme sensitive end of the sulfur response distribution were homozygous for sulfur sensitivity alleles at all three QTL and had mean damage of 68.6 and 67.3% for cotyledons and leaves, respectively.

The distribution of sulfur tolerance in the population did not fit the assumptions of the MQM methods so non-parametric interval mapping was used to confirm QTL. Non-parametric QTL mapping of sulfur tolerance means of cotyledons and leaf tissue both across and within tests verified the association of qSulf-1 in all instances but found no minor QTL.

### Marker Development

The major QTL qSulf-1 was identified in an area of the map with low SNP density, and collocated with only two binned SNPs. The remaining 157 kb did not have any SNPs from the GBS data. The closest SNPs flanking the QTL peak were 0.9 and 1.3 cM away. Therefore, the first objective for KASP design was to saturate the region to increase resolution of qSulf-1. The second objective was to design markers at regular intervals with decreasing frequency as distance from the QTL peak increased that would be able to track the breakage of linkage drag for future marker-assisted backcross selection. SNPs were identified and chosen for design from whole-genome resequencing data of the parents of the population, MR-1 and AY. Paired-end libraries were sequenced, generating 52.3 million reads for the sulfur tolerant parent (AY) and 44.5 million reads for the sulfur sensitive parent (MR-1). An initial set of 3.3 million SNPs was called between the parental genomes and then filtered to 304,864 SNPs. While the qSulf-1 interval had 154 SNPs between the parents, 18 were chosen to fill gaps in the original genetic map. Genotypes of the RIL population from the KASP markers were used for QTL mapping both alone and in combination with the original GBS population genotypes.

**Figure 5:**
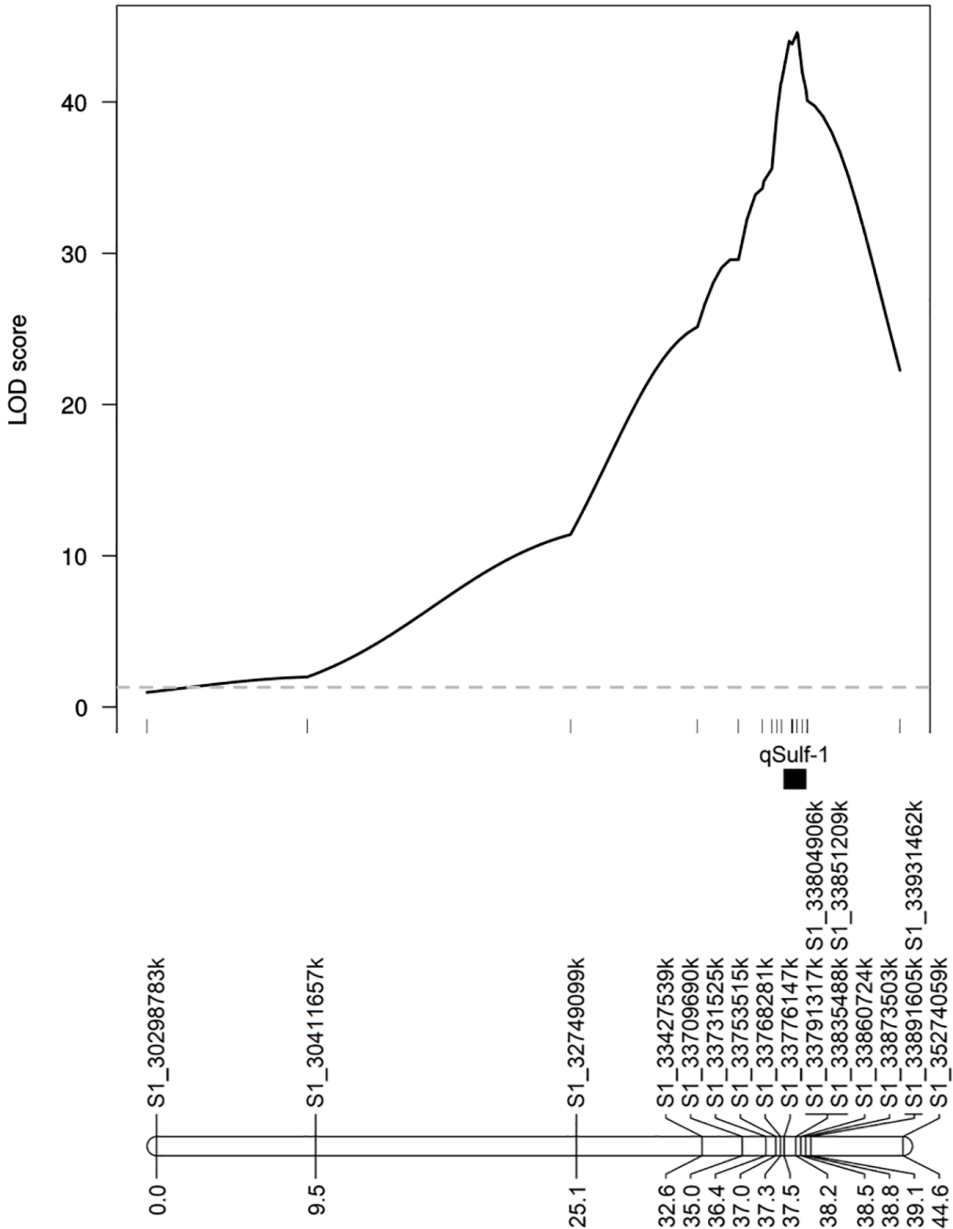
Logarithm of odds (LOD) scores of the association of 18 KASP markers with mean percentage of damaged (chlorotic or necrotic) leaf area after vaporized elemental sulfur treatment. Genetic positions of the markers and the QTL (qSulf-1) are shown below the plot.

MQM using the 18 KASP markers identified the same QTL peak for both tissue types, located at 33,860,724 bp on chromosome 1 (**Figure 5**). Seven KASP markers were located in the 1.5-LOD interval of the qSulf-1 with an average genetic distance between markers of 0.20 cM. The remaining markers were located outside of the QTL region with frequency decreasing with genetic distance from the peak of qSulf-1 (**Figure 5**).

MQM of sulfur tolerance using the combined genotypic dataset (GBS and KASP; N = 5,681 SNPs) improved saturation and resolution of qSulf-1 for both tissue types. The physical distance of the 1.5-LOD interval decreased by 141 kb for cotyledons and 59 kb for leaf tissue (**Table 2**). The narrowed interval corresponded to the exact same physical positions for both tissue types, which extended from 33,776,147 to 33,873,503 bp (97,356 bp) on chromosome 1. In addition, qSulf-12 surpassed the significance threshold for the leaf phenotype in the combined genotypic dataset while it had not with GBS SNPs alone (**Table 2**).

Five KASP markers were tightly linked to qSulf-1 in the RIL population, including the peak LOD score of 49.54 at 190.74 cM (Sulf1_33860724) and a haplotype block of 4 SNPs (Sulf1-33791317, Sulf1-33804906, Sulf1-33835488 and Sulf1-33851209) that map 0.01 cM away (190.73 cM) with a LOD score of 49.46. The mean percent of affected leaf area for RILs homozygous for the sulfur tolerance allele at each of these SNPs was 6%.

The KASP markers were used to genotype thirty melon accessions to determine whether these markers could be successfully utilized in a variety of breeding programs (**Supplementary Table S3**). Nine of the eighteen markers (bolded in **Supplementary Table S3**) were significantly associated with the sulfur response of the accessions. Two markers had the strongest association, Sulf1-33791317 (R2 = 0.66) and Sulf1-33835488 (R2 = 0.72), but each had one sensitive accession with homozygous tolerant alleles. Both markers were also among the most tightly linked to sulfur response in the RIL population.

### Candidate Genes

Candidate genes for sulfur tolerance in melon were identified by comparing the 1.5-LOD interval of qSulf-1 (GBS + KASP) with the physical location of the binned SNPs in the *C. melo* reference genome. Twenty-one genes (**Supplementary Table S4**) were encoded in the qSulf-1 interval (33,776,147 to 33,873,503 bp on chromosome 1). Whole genome sequencing identified 129 SNPs and 56 indels between the parents in the qSulf-1 region. Functional annotation of the polymorphisms narrowed the potential candidates to eight genes that had mutations most likely to cause regulatory or structural changes to the resulting proteins (**Supplementary Table S5**). Seven genes had SNPs or indels in the promoter region which may alter their expression levels. MELO3C024245 had two missense mutations in exon 5, altering the resulting amino acid sequence (**Supplementary Table S5**).

## Discussion

The strong skew towards tolerance in the response of the RIL population to vaporized elemental sulfur can be explained by the epistatic interactions of the genes contributing to its polygenic inheritance. One major (qSulf-1) and two minor QTL (qSulf-8 and qSulf-12) were associated with sulfur tolerance in this study. RILs homozygous for the sulfur tolerance allele at the major QTL (qSulf-1) exhibited low damage independent of their genotype at the other loci. RILs homozygous for sulfur sensitivity alleles at qSulf-1 and tolerance alleles at one or both minor QTL displayed intermediate tolerance. RILs with sulfur sensitivity alleles at all three loci formed the extreme tail of the distribution.

Perchepied et al. (2004) found inheritance of sulfur tolerance to be polygenic in a QTL mapping study of sulfur tolerance in two sets of melon RILs with a common tolerant parent (‘Vedrantais’) and different sensitive parents (PI 161375 and PI 124112). Although the physical position of qSulf-1 cannot be compared to the QTL from this study, as the reference genome was not yet available, the chromosome names of the reference genome were based upon those of the linkage groups of the genetic map (Périn et al., 2002) used by Perchepied et al. (2004). Both studies found one major QTL on the proximal end of chromosome 1 with a similar contribution to variation in sulfur damage. Perchepied et al. (2004) also identified two minor QTL in one of the populations but they were found on different chromosomes than qSulf-8 and qSulf-12.

The limited research on tolerance to sulfur phytotoxicity in melon has focused on sulfur dust application for screening and QTL discovery (Johnson and Mayberry, 1980; Perchepied et al., 2004). Extensive research on the susceptibility of cucurbits to oxidized and reduced forms of sulfur may provide indications of the underlying mechanism of sulfur tolerance in some melon lines. Plants take in sulfur through their roots as sulfate and through their leaves primarily as sulfur dioxide and hydrogen sulfide, but excess sulfur accumulation can become toxic at levels that vary by species, varieties, soil-sulfur content, and environmental conditions (Rennenberg, 1984; Hawkesford and De Kok, 2006). Hydrogen sulfide can be oxidized by the plant to sulfate and reintroduced into the sulfur reduction pathway (Rennenberg and Filner, 1982), which through a series of enzymatic reactions produces cysteine, methionine, and glutathione (Hawkesford and De Kok, 2006). RILs in the melon population described in this report that are tolerant to sulfur toxicity may utilize the same pathways to expel excess elemental sulfur. One of the potential candidate genes based upon function includes a receptor-like protein kinase (RLK; MELO3C024237) that had eight polymorphisms in the promoter region. RLKs have been shown to play a critical role in plant responses to abiotic stresses in many studies (reviewed in Ye et al., 2017). A potential mechanism of sulfur tolerance may be that the RLK activates an enzyme in the sulfur metabolism pathway but lower expression of the RLK in MR-1 limits catabolism and sulfur accumulates. Ferredoxins provide the elections for sulfite reductase to reduce sulfite to sulfide, the precursor substrate for all products of the pathway (Hell, 1997). A gene encoding a ferredoxin (*MELO3C024264*) collocated with the original (GBS alone) qSulf-1 interval but was just outside the significant boundary after the addition of KASP markers. The sulfur sensitivity contributed by the MR-1 allele could be caused by lower expression of ferredoxin (due to an indel in the promoter region) limiting sulfur metabolism resulting in a buildup of sulfur to toxic levels.

MR-1 is known to be highly resistant to powdery mildew (Thomas, 1986), downy mildew (Thomas, 1986), Fusarium wilt (Branham et al., 2018), and *Alternaria* leaf blight (Thomas et al., 1990; Daley et al., 2017), making it an excellent source for resistance breeding. However, MR-1 is highly susceptible to both powdered and volatilized sulfur, thus introduction of sulfur susceptibility into a normally sulfur resistant elite line is a real concern. Here, we provide KASP markers tightly linked to the major sulfur tolerance QTL (qSulf-1), which can immediately be incorporated into melon breeding programs. These KASP markers will allow breeders to incorporate the disease resistances of MR-1 without the inadvertent introgression of sulfur susceptibility. MR-1 has poor horticultural quality in all traits (brix, texture, cracking, shape, etc.), therefore most breeding programs are likely to incorporate its many disease resistance alleles through backcrossing to an elite cultivar. Five of the markers released here are tightly linked to sulfur sensitivity in MR-1, while the remaining thirteen flank qSulf-1 with decreasing frequency. These markers will allow efficient tracking of introgression to limit linkage drag while ensuring exclusion of the major sulfur sensitivity allele of MR-1. In addition, two of the markers were significantly associated with sulfur response in a diverse set of cultivars suggesting they could be used to both avoid inadvertent introgression of sulfur sensitivity and introduce sulfur tolerance dependent upon the breeding materials chosen.

## Data Availability Statement

The datasets generated for this study can be found in the Dryad Data Repository, doi: 10.5061/dryad.zkh18937m.

## Ethics Statement

The experiment conducted complies with the laws of the United States.

## Author Contributions

WW designed and implemented the experiments. JD optimized the sulfur protocols. WW and SB phenotyped the population. SB analyzed the data. SB, WW, AL, RH, and JD wrote the manuscript. All authors contributed to the article and approved the submitted version.

## Funding

This study was funded, in part, by the United States Department of Agriculture (USDA) project number 6080-22000-028-00 and the National Institute of Food and Agriculture, Specialty Crops Research Initiative project number 6080-21000-019-08.

## Conflict of Interest

The authors declare that the research was conducted in the absence of any commercial or financial relationships that could be construed as a potential conflict of interest.

## Supplementary Material

Supplementary Table S1 Sequence information for the KASP markers, including: SNP ID, physical position of the SNP, primer sequences, SNP flanking sequence, and nucleotides of the sulfur tolerant (T) and sensitive (S) alleles.

Supplementary Table S2 Phenotypic data used for QTL mapping: RIL means of percentage of damaged (chlorotic or necrotic) area after vaporized elemental sulfur treatment of cotyledons (cot) or leaves (leaf) in test 1 (_t1), in test 2 (_t2), and across tests (cot or leaf).

Supplementary Table S3 Melon accession genotypes at KASP markers developed across qSulf-1. Markers are named according to physical position (bp) on chromosome 1. Genotypes are color coded, with individuals homozygous for the sulfur tolerance allele (**B**) in blue, sensitivity allele (**A**) in yellow, heterozygous (**H**) in gray and missing (**NA**) in white. The significance and magnitude of correlation between sulfur response and genotype are listed for each marker

Supplementary Table S4 iChromosomal location and functional nformation for genes that collocated with the major QTL for sulfur tolerance, including the position of the start and stop codons within the chromosome (cs), gene ontology (GO) code and term, and conserved domains and features found within the gene (NCBI).

Supplementary Table S5 Functional annotation of candidate gene polymorphisms between the parents in the QTL interval of qSulf-1, including the chromosome, physical position (in bp), parental alleles, polymorphism location relative to the gene (ie. upstream, exonic, downstream, etc.), distance from the gene (in bp), type of exonic mutation (synonymous or nonsynonymous), and detail (which exon and the nucleotide and amino acid changes).

Supplementary Figure S1 Interaction plot showing evidence for epistasis between qSulf-1 and qSulf-12. Alleles from the sulfur sensitive parent (MR-1) are ‘AA’ and from the sulfur tolerant parent (AY) are ‘BB’. The circle represents the mean percent damage of individuals in the population with the labelled genotypes. The plus signs indicate the standard error.

Supplementary Figure S2 Logarithm of odds (LOD) scores for forward model selection of up to seven QTL associated with mean percentage of damaged (chlorotic or necrotic) area after vaporized elemental sulfur treatment of: (**a**) cotyledons in test 1, (**b**) cotyledons in test 2, (**c**) leaves in test 1 and (**d**) leaves in test 2. The initial scan shows the likelihood of the first QTL being located at each SNP in the genome (linkage group=chromosome) with subsequent scans showing the LOD of an additional QTL with the effects of the previous QTL(s) controlled for in the model. The dashed line marks the genome-wide significance threshold.

Supplementary Figure S3 Interaction plot showing evidence for epistasis between qSulf-1 and qSulf-8. Alleles from the sulfur sensitive parent (MR-1) are ‘AA’ and from the sulfur tolerant parent (AY) are ‘BB’. The circle represents the mean percent damage of individuals in the population with the labelled genotypes. The plus signs indicate the standard error.
