# Supplementary figures and images for: QTL Mapping and Marker Development for Tolerance to Sulfur Phytotoxicity in Melon (Cucumis melo)
Source: Front Plant Sci. 2020 Jul 22;11:1097. doi: 10.3389/fpls.2020.01097 (PMC7387513; doi:10.3389/fpls.2020.01097)

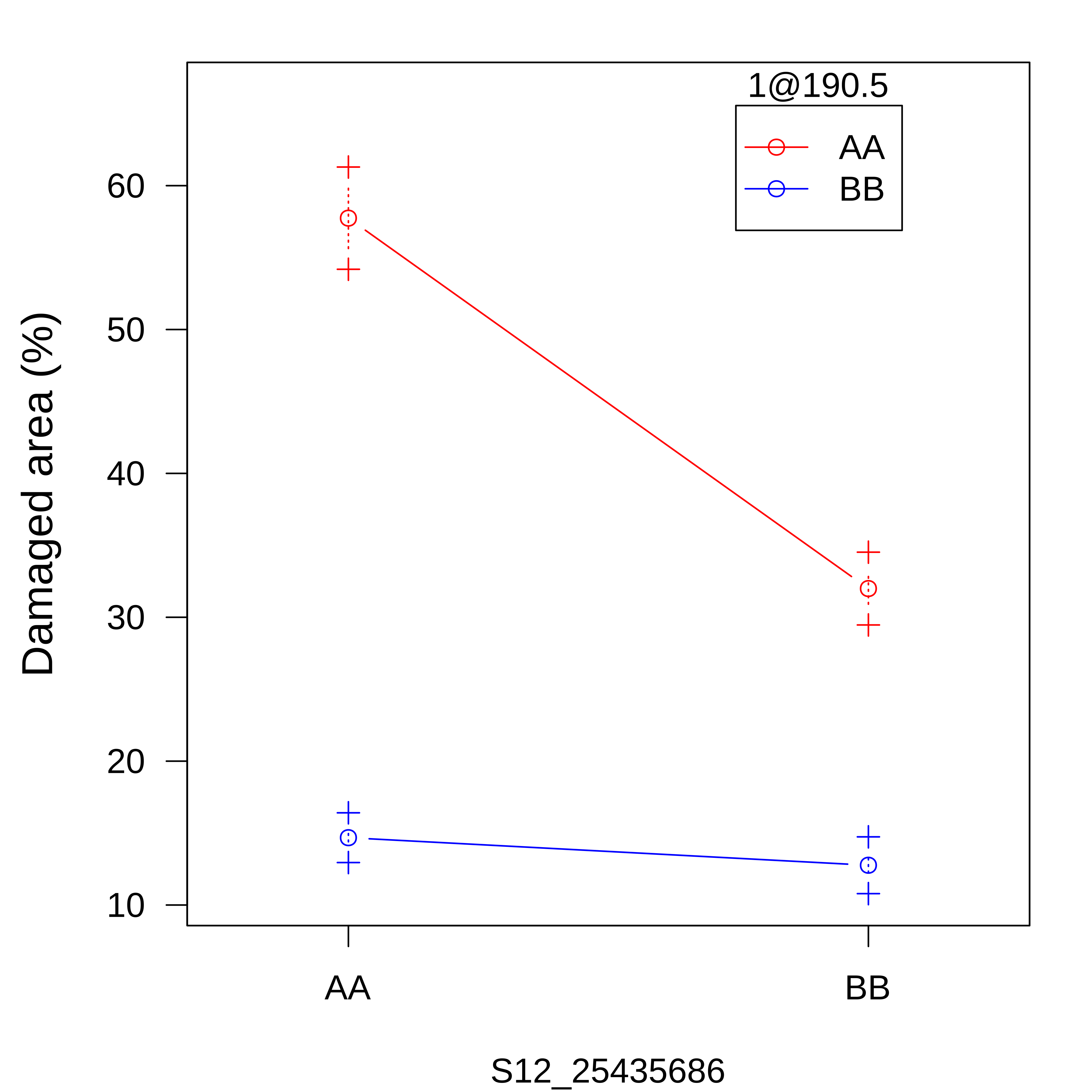

Supplement: Supplementary Figure S1 — Interaction plot showing evidence for epistasis between qSulf-1 and qSulf-12. Alleles from the sulfur sensitive parent (MR-1) are ‘AA’ and from the sulfur tolerant parent (AY) are ‘BB’. The circle represents the mean percent damage of individuals in the population with the labelled genotypes. The plus signs indicate the standard error. [file Image_1.tif]

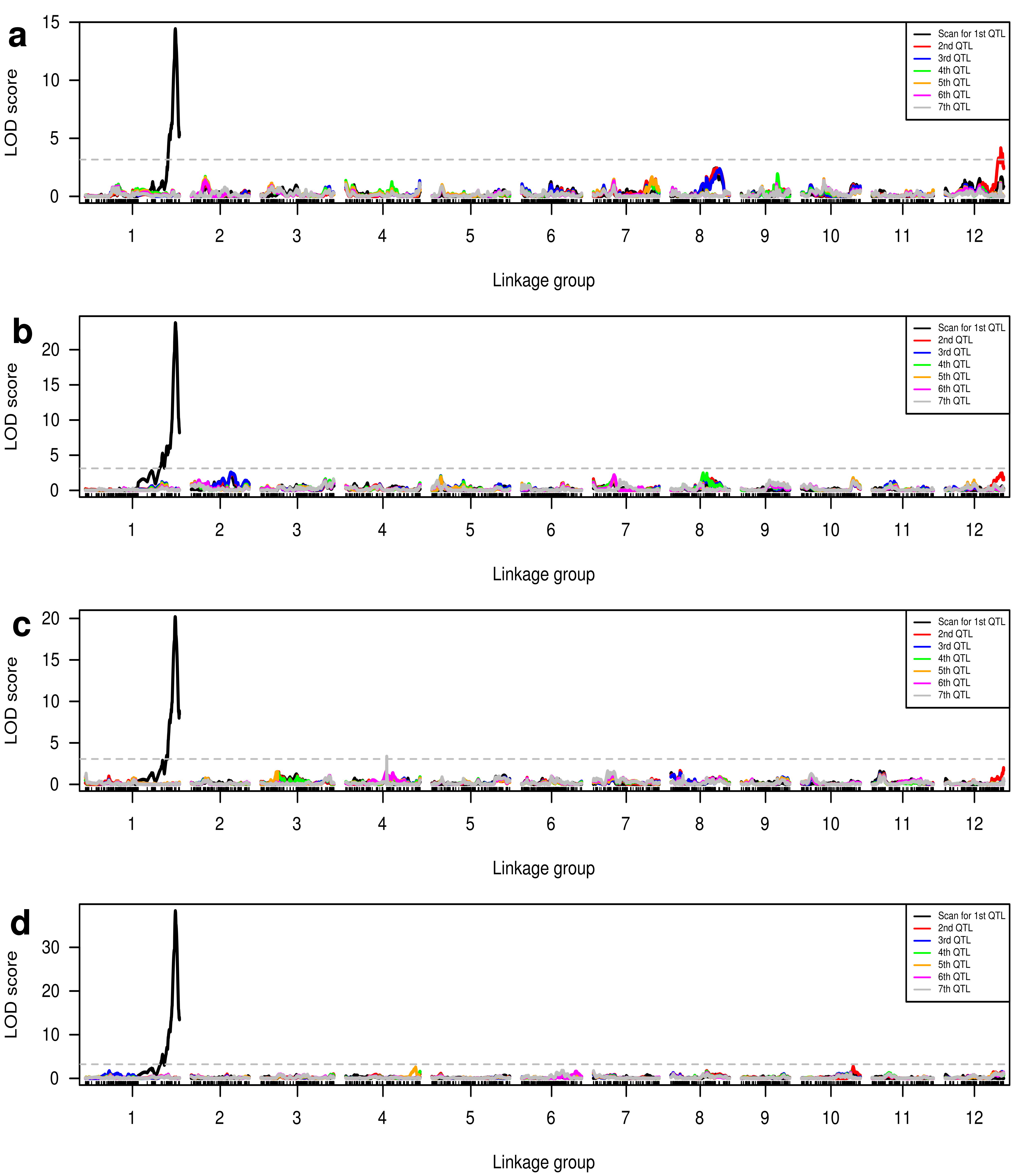

Supplement: Supplementary Figure S2 — Logarithm of odds (LOD) scores for forward model selection of up to seven QTL associated with mean percentage of damaged (chlorotic or necrotic) area after vaporized elemental sulfur treatment of: (a) cotyledons in test 1, (b) cotyledons in test 2, (c) leaves in test 1 and (d) leaves in test 2. The initial scan shows the likelihood of the first QTL being located at each SNP in the genome (linkage group=chromosome) with subsequent scans showing the LOD of an additional QTL with the effects of the previous QTL(s) controlled for in the model. The dashed line marks the genome-wide significance threshold. [file Image_2.tif]

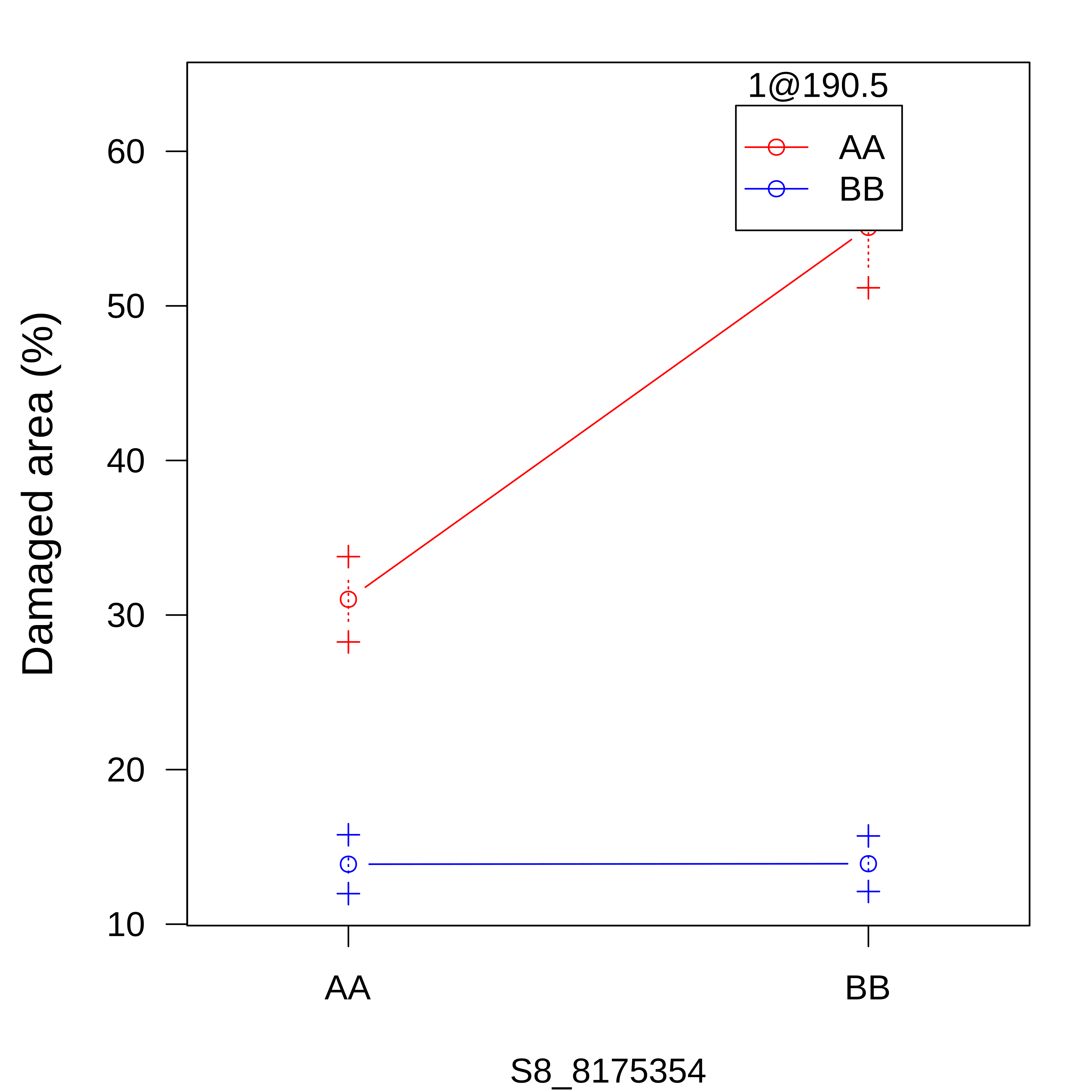

Supplement: Supplementary Figure S3 — Interaction plot showing evidence for epistasis between qSulf-1 and qSulf-8. Alleles from the sulfur sensitive parent (MR-1) are ‘AA’ and from the sulfur tolerant parent (AY) are ‘BB’. The circle represents the mean percent damage of individuals in the population with the labelled genotypes. The plus signs indicate the standard error. [file Image_3.tif]
